# Prevalence and multilocus genotyping of *Giardia duodenalis* in pigs of Shaanxi Province, northwestern China

**DOI:** 10.1186/s13071-017-2418-8

**Published:** 2017-10-17

**Authors:** Sha-Sha Wang, Ya-Jie Yuan, Yan-Ling Yin, Rui-Si Hu, Jun-Ke Song, Guang-Hui Zhao

**Affiliations:** College of Veterinary Medicine, Northwest A&F University, 22 Xinong Road, Yangling, 712100 People’s Republic of China

**Keywords:** *Giardia duodenalis*, Prevalence, MLG, Pig, Shaanxi Province, China

## Abstract

**Background:**

Giardiasis, caused by *Giardia duodenalis* (syn. *Giardia intestinalis*, *Giardia lamblia*), is a significant zoonotic parasitic disease of animals and humans worldwide. Accurate genotyping of *G. duodenalis* is essential for efficient control and management of giardiasis. The objectives of the present study were to investigate the prevalence and assemblages of giardiasis in pigs in Shaanxi Province, northwestern China, and for the first time study multilocus genotypes (MLGs) in pigs using multilocus genotyping technology in this region.

**Results:**

Of 560 faecal samples collected from five farms in Shaanxi Province, 45 were positive for *G. duodenalis* and significant differences in prevalence were observed among different locations. Differences in prevalence were also detected in pigs of different age groups, with the highest prevalence in sows and the lowest in boars. Two assemblages, A and E, were identified, and a mixed infection of both A and E was identified in one faecal sample. Assemblage E was predominant and widely distributed in all investigated areas and age groups. Genetic viability was detected for both assemblages, and four different multi-locus genotypes (MLGs) within assemblage E were found, MLGE1-MLGE4.

**Conclusions:**

*Giardia duodenalis* was detected in pigs from Shaanxi Province, northwestern China, and genetic diversity was observed in these infections. Both assemblages A and E were detected, and four distinct MLGs within assemblage E were identified. These findings provide new data for controlling *G. duodenalis* infection in pigs.

## Background

*Giardia duodenalis* (syn. *Giardia intestinalis*, *Giardia lamblia*), an important parasitic protozoan, inhabits the gastrointestinal tracts of animals. It causes giardiasis, with clinical presentations ranging from chronic to acute diarrhea, dehydration, abdominal pain, nausea, vomiting, and weight loss [1], leading to large economic impacts [2]. Giardiasis is mainly transmitted through the faecal-oral route (e.g. water or food) [3]. The public health impact of giardiasis is significant because of its tendency to cause major outbreaks and its adverse effects on growth and cognitive functions in children [4, 5]. *Giardia duodenalis* has also been reported in a wide variety of other hosts worldwide, including sheep, goats, cattle, and non-human primates [6–20].

Recent molecular analysis indicated eight major morphologically similar but genetically distinct assemblages of *G. duodenalis*, assemblages A-H [21]. Among them, assemblages A and B have been identified in both humans and animals [13], whereas the remaining six assemblages (C–H) infect non-human hosts; however, assemblages C, D, E, and F have also been identified in humans [2, 22].

In China, *G. duodenalis* has been identified in sheep (4.3–6.6%) [15, 16], goats (2.9–12.7%) [16, 17] and cattle (1.1–60.1%) [19–21]. Although most infections were asymptomatic, cysts excreted in faeces could be a possible source of infection for humans and other animals [23]. Pigs are an economically important food animal, providing pork to many nations, and pig manure is sometimes used in the cultivation of food and feed crops [24]. *Giardia duodenalis* infection has been reported in pigs in many countries (Table 1), with the zoonotic assemblages A and B have been detected in pigs [25], suggesting that pigs may be a reservoir of human infection. China is recognized as the largest pig breeding country in the world, with about 667 million pigs produced annually, however, prior to the present study, no public reports on *G. duodenalis* infection in pigs of China were available.

**Table 1:** Global prevalence of *Giardia duodenalis* infection in pigs

Country (City)	No. examined	Prevalence (%)	Locus	Detection method	Time tested (year)	Reference
Australia (unknown)	289	31.1	SSU rRNA	PCR	2005–2006	[41]
Canada (Edward)	633	1.0	SSU rRNA, *bg*	Immunofluorescence microscopy and PCR	2007	[48]
Canada (Ontario)	122	66.4	SSU rRNA, *bg*	Immunofluorescence microscopy and PCR	2005–2006	[42]
Canada (unknown)	236	9.0	–^a^	Immunofluorescence microscopy	1995	[54]
Cambodia (Preah Vihear)	76	0	SSU rRNA	Immunofluorescence microscopy and PCR	2012	[49]
China (Shaanxi)	560	8	*bg*, *tpi*, *gdh*	PCR	2016–2017	This study
Denmark (unknown)	1237	17.4	–^a^	Immunofluorescence microscopy	2003–2004	[43]
Denmark (unknown)	856	14.0	SSU rRNA, *gdh*	Immunofluorescence microscopy and PCR	2011–2012	[44]
Denmark (unknown)	1237	17.4	SSU rRNA, *gdh*	PCR	2003–2004	[45]
Turkey (Istanbul)	238	3.7	– ^a^	Immunofluorescence microscopy	2005	[50]
Norway (unknown)	684	1.5	SSU rRNA	Immunofluorescence microscopy and PCR	2004–2005	[51]
Poland (unknown)	84	9.5	*bg*	Immunofluorescence microscopy and PCR	2013–2014	[46]
UK (Preston, Cheshire)	7	57.1	SSU rRNA	PCR	2007–2008	[47]
USA (Ohio)	325	7.4	–^a^	Immunofluorescence microscopy	1993	[52]
Zambia (Lusaka)	217	12.0	–^a^	Immunofluorescence microscopy	2011	[25]

^a^PCR not used to amplify gene locus

Previous studies to investigate *G. duodenalis* used morphological methods or molecular technologies based on one or two gene loci (Table 1). Morphological examination is time- and labor-consuming, and cannot identify assemblages [26]. Molecular assay using one or two gene loci could not differentiate mixed infectious and did not provide sufficient information to understand the possible zoonotic links [27]. Recently, a multilocus genotyping technique was developed and has been applied to genotype *G. duodenalis* in dairy calves [28], native beef calves [20], sheep [15], raccoon dogs [29], children [30], pet chinchillas [31], red deer, roe deer [32] and other hosts [33]. Using four gene loci, namely β-giardin (*bg*), glutamate dehydrogenase (*gdh*), triosephosphate isomerase (*tpi*) and the small subunit ribosomal RNA (SSU rRNA), several multilocus genotypes (MLGs) and mixed genotypes were observed, including one MLGA and four MLGE in dairy calves [28], one MLGA, twenty-two MLGE and two mixed A + E in native beef calves [20], one MLGA, six MLGE and three mixed in sheep [15], three MLGC in raccoon dogs [29], two MLGA and three MLGE in pet chinchillas [31], and two MLGA and nine MLGE in children [30]. The objectives of the present study were to determine the prevalence and assemblages of *G. duodenalis* in pigs in Shaanxi Province, northwestern China, and investigate the MLGs in pigs using multilocus genotyping tool.

## Methods

### Sample collection

**Fig. 1:**
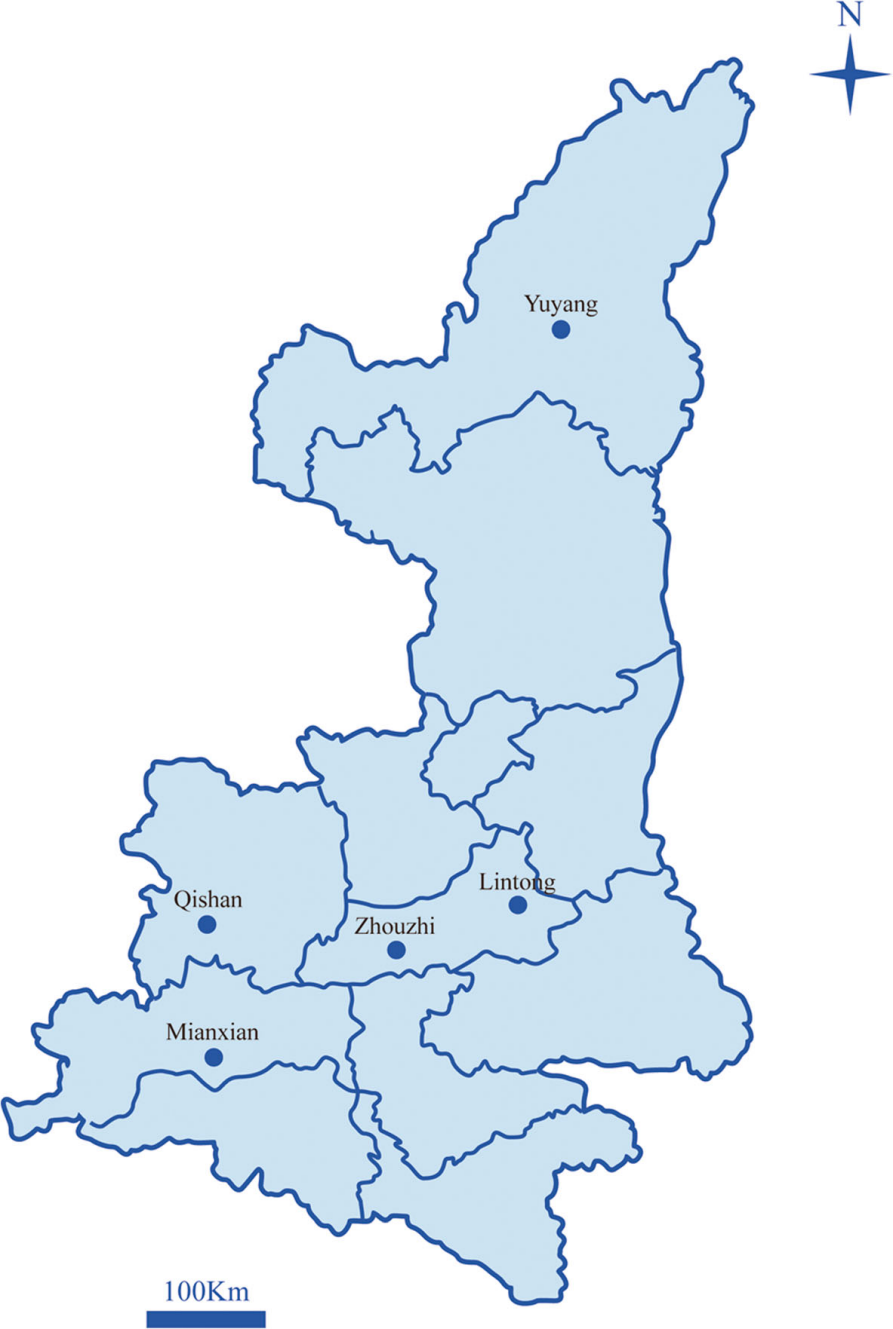
Sampling sites in this study

Shaanxi Province is located across the Qinling Mountains, which is the border between the North and South of China. It has gradually become one of the important regions of the pig industry due to environmental pollution and disease epidemics in the traditional pig breeding areas in northern China. In 2016, there were 3901 large pig farms operating in Shaanxi Province. In order to determine the prevalence and assemblage distribution of *G. duodenalis* in pigs in Shaanxi Province, northwestern China, 560 faecal samples were collected from pigs (newborn to 2 years) from five different farms in Zhouzhi, Qishan, Mianxian, Lintong and Yuyang, between September 2016 and March 2017 (Fig. 1). The 560 faecal samples comprised samples from suckling piglets aged < 25 days, weaned piglets aged 1–4 months, fatteners aged 4–6 months, and sows and boars aged 6 months to 2 years. Fresh normal faeces were randomly sampled from apparently healthy pigs of all age groups and for whom antibiotics or other antimicrobials were not used. Samples were placed into individual sterile plastic containers, marked with the geographical origin, date, breed, age and sample number. All faecal samples were then transported immediately to the laboratory on ice packs, preserved in 2.5% potassium dichromate and stored at 4 °C for further analysis.

### Genomic DNA extraction

Each faecal sample was washed three times in distilled water with centrifugation at 13,000 *rpm* for 1 min to remove the potassium dichromate. Genomic DNA of each sample was extracted from approximately 300 mg of washed faecal material, using the commercial E.Z.N.A® Stool DNA kit (Omega Bio-Tek Inc., Norcross, GA, USA), according to the manufacturer’s instructions. Extracted DNA samples were stored at -20 °C prior to PCR analysis.

### Nested PCR amplification

The prevalence of *G. duodenalis* in pigs was initially determined by nested PCR targeting the *bg* gene fragment using primers described previously [34] in a 25 μl PCR mixture containing 1 μl genomic DNA (for the primary PCR) or 1 μl of the primary amplification product (for the secondary PCR) as the template, 2.5 μl 10× *Ex Taq* Buffer (Mg^2+^ free), 2 mM MgCl_2,_ 0.2 mM dNTP Mixture, 0.625 U of TaKaRa *Ex Taq* (TaKaRa Shuzo Co., Ltd) and 0.4 μM of each primer (Table 2).

**Table 2:** PCR primers used in this study

Gene locus	Primer name	Sequence (5'-3')	Amplicon length (bp)	Annealing temperature (°C)	Reference
*bg*	G7-F	TCAACGTYAAYCGYGGYTTCCGT	573	52	[35]
	G759-R	CAGTACACCTCYGCTCTCGG			
	G99-F	GAACGAACGAGATCGAGGTCCG	511	55	
	G609-R	CTCGACGAGCTTCGTGTT			
*tpi*	AL3543	AAATIATGCCTGCTCGTCG	605	50	[34]
	AL3546	CAAACCTTITCCGCAAACC			
	AL3544	CCCTTCATCGGIGGTAACTT	530	58	
	AL3545	GTGGCCACCACICCCGTGCC			
*gdh*	GDHeF	TCAACGTYAAYCGYGGYTTCCGT	432	52	[35]
	GDHeR	GTTRTCCTTGCACATCTCC			
	GDHiF	CAGTACACCTCYGCTCTCGG	432	65	
	GDHiR	GTTRTCCTTGCACATCTCC			

To investigate multi-locus genotypes (MLGs) of *G. duodenalis* in pigs, the *bg*-positive samples were then amplified using primers for the *gdh* and *tpi* gene loci described previously [34, 35] (Table 2). The PCR products were then examined by electrophoresis in 1% (*w*/*v*) agarose gels with ethidium bromide staining.

### Sequencing and sequence analysis

All positive PCR products were sent to Xi’an Qingke Biological Co., Ltd. for direct sequencing on an ABI PRISM 3730 XL DNA Analyzer (Applied Biosystems, Foster City, CA, USA) using relevant internal nested primers for PCR amplification. Sequences obtained were aligned with sequences available on GenBank using Basic Local Alignment Search Tool (BLAST), and edited using DNAStar 5.0 [36] and Clustal X 1.81 [37]. *Giardia duodenalis* assemblages were identified by their alignment to reference sequences available from GenBank. MLGs were identified for samples which were successfully sequenced at all three loci.

### Statistical analysis

Chi-square (*χ* ^2^) analysis and 95% confidence intervals (CIs) were calculated using SPSS 19.0 for Windows (SPSS Inc., Chicago, IL, USA) and used to analyze differences between different locations and age groups, with *P* < 0.05 considered statistically significant.

### Nucleotide sequence accession numbers

All nucleotide sequences obtained in this study were submitted to the National Center for Biotechnology Information (NCBI) GenBank database under the following accession numbers: KY989575–KY989579 for the *bg* gene, KY989580–KY989583 for the *tpi* gene, and MF034655–MF034658 for the *gdh* gene.

## Results and discussion

Globally, *Giardia duodenalis* is one of the most common intestinal parasites in symptomatic and asymptomatic humans and livestock [38]; this species is relatively common in pigs worldwide (Table 1). Although no clinical signs are observed in most pigs carrying *G. duodenalis*, they still shed infective *G. duodenalis* cysts into the environment which can survive for extended periods in cool, humid environments. Considering that exposure to infective cysts through contaminated water and food is the primary mechanism of *G. duodenalis* transmission to animals and humans [39, 40], investigating *G. duodenalis* infection in pigs has important implications for controlling giardiasis in humans and animals.

Varying prevalence rates of *G. duodenalis* have been reported in livestock in China, e.g. 4.3–6.6% in sheep [14–16], 2.9–12.7% in goats [16, 17] and 1.1–60.1% in cattle [18–20]. In the present study, of the 560 faecal samples examined from five locations, 45 (8%, 95% CI: 7.4–8.7%) were positive for *G. duodenalis* infection (Table 3). Significantly different (*χ* ^2^ = 28.514, *df* = 4, *P* < 0.0001) prevalences were observed among different locations, with the highest (16.7%, 17/102) in Lintong district and the lowest (1.0%, 1/100) (*χ* ^2^ = 13.909, *df* = 1, *P* < 0.01) in Qishan county. Comparison of these results with results obtained from other pig farms showed that the prevalence of *G. duodenalis* in pigs in Shaanxi Province in China was lower than that in Australia (31.1%) [41], Ontario, Canada (66.4%) [42], Denmark (14.0–17.4%) [43–45], Poland (9.5%) [46], Lusaka, Zambia (12.0%) [25], and Preston and Cheshire, UK (57.1%) [47], but higher than in Prince Edward Island, Canada (1.0%) [48], Preah Vihear, Cambodia (0) [49], Istanbul, Turkey (3.7%) [50], Norway (1.5%) [51] and Ohio, USA (7.4%) [52]. The differences are probably due to a range of factors, including the presence of other animal species on the farm, examination methods, study design, number of samples analysed, time of specimen collection, environmental conditions and farm management practices [28, 53]. For example, slightly higher prevalences were observed from some pig farms with multiple animal species raised in the same farms (e.g. 57.1% in the UK) (Table 1). In our study, two farms from Mianxian and Lintong also housed dogs and ducks, and the prevalence of *G. duodenalis* was comparatively higher (9.0% and 16.7%, respectively). These findings could suggest transmission between the different animals, which should be explored in future studies.

**Table 3:** Prevalence and factors associated with *G. duodenalis* infection in pigs in Shaanxi Province, northwestern China

Variable	Category	No. examined	No. positive (%)	Target locus (no. positive)		
				*bg*	*tpi*	*gdh*
Age	Suckling piglest	155	10 (6.5)	10	4	5
	Weaned pigs	220	20 (9.1)	20	8	4
	Fatteners	98	8 (8.2)	8	6	2
	Sow	57	6 (10.5)	6	2	0
	Boar	30	1 (3.3)	1	0	0
	Total	560	45 (8.0)	45	20	11
Location**	Zhouzhi county	143	2 (1.4)	2	1	0
	Qishan county	100	1 (1.0)	1	0	0
	Mianxian county	100	9 (9.0)	9	5	3
	Lintong district	102	17 (16.7)	17	12	4
	Yuyang district	115	16 (13.9)	16	2	4
	Total	560	45 (8.0)	45	20	11

**P < 0.0001

Differences in *G. duodenalis* prevalence were detected in pigs of different age groups in this study, but these differences were not statistically significant (*χ* ^2^ = 2.056, *df* = 4, *P* > 0.05). The highest prevalence (10.5%, *χ* ^2^ = 1.264, *df* = 1, *P* > 0.05) was detected in sow pigs, which was consistent with a study from Zambia (53.3%) [25], but was different to a study performed in Australia (30.0%) [41] and one study from Denmark (14.0%) [44], where the highest prevalence was found in weaned pigs. The second highest prevalence (9.1%) was observed in weaned pigs and the lowest infection rate was found in boars, with a prevalence of 3.3%, which was different with a study in Zambia [25], in which the sucking piglets had the lowest infection rate (25%). Although previous studies have suggested that the immunity, nutritional status, geographical separation and gut microbiome could contribute to the variable prevalence in pigs of different age groups [44], the actual association between pig age and *G. duodenalis* infection should be further evaluated in future studies.

Genetic variability of *G. duodenalis* has been reported in pigs and five assemblages (A, B, D, E, F) have been reported [41, 42, 44–46]. In the present study, two assemblages, A and E, were detected among 45 *G. duodenalis*-positive samples based on the *bg* gene, with assemblage E (80%, 36/45) being the predominant assemblage, which was detected in all investigated areas and age groups. These results were consistent with a study in Australia [41] and two studies from Denmark [44, 45]. Additionally, the highest prevalence of the assemblage E was observed in weaned pigs in our study and studies in Denmark [44] and Australia [41]. While assemblage A (20%, 9/45) was only found in pigs from Zhouzhi county, Lintong district and Yuyang district, it was widely distributed in all age groups except boars. The reason from the higher prevalence of assemblage A in these specific locations is worthy of further investigation. Comparison with previous studies [41, 42, 44, 45] also indicated that this was the first report for assemblage A in sow.

To further illuminate the genetic diversity of *G. duodenalis* in pigs, the sequence characters of the *tpi* and *gdh* genes were analyzed for the 45 *bg* positive samples and the MLGs were characterized in pigs using combined data from these three gene loci. Of 45 *bg*-positive samples, 9 *tpi* and 11 *gdh* gene sequences were obtained. Sequence alignment identified different genotypes of assemblages E (Table 4) and A (Table 5). Eight faecal samples of assemblage E were successfully sequenced at all three gene loci, forming four different assemblage E MLGs, named as MLGE1-MLGE4 (Table 6). MLGE1 and MLGE4 were only found in weaned pigs from Mianxian county and fatteners from Yuyang district, respectively. Both MLGE2 and MLGE3 were detected in pigs from Lintong district, but they were distributed in different age groups, with MLGE2 in suckling pigs and MLGE3 in both weaned pigs and fatteners. Although no zoonotic assemblage A MLGs were obtained in our study, a mixed assemblage of E and A infections was found in one isolate (LTD6) from fatteners in Lintong district, which would be the result of mixed infection or genetic exchange between assemblages [20]. Previous studies also detected mixed infections of these two assemblages in pigs from Denmark based on *gdh* and SSU rRNA sequences [44] and other reports using *bg*, *gdh*, *tpi*, and SSU rRNA sequences in dairy calves [28], dairy cattle [20], and sheep [15]. This suggests that multilocus genotyping would be an accurate tool to determine mixed infections, zoonotic potential and genetic variability of *G. duodenalis* in animals as well as humans.

**Table 4:** Intra-assemblage substitutions in *bg*, *tpi* and *gdh* sequences from assemblage E

Subtype (number)	Nucleotide positions and substitutions				GenBank ID
	57	120	180		
*bg*					
Ref. sequence	T	C	C		KU668892
E (36)	T	C	C		KY989575
	56	143	340		
*tpi*					
Ref. sequence	C	C	C		KJ668136
E1 (6)	C	C	C		KY989581
E2 (8)	C	T	C		KY989580
E3 (1)	C	C	T		KY989582
	68	216	285	303	
*gdh*					
Ref. sequence	T	T	C	C	JN160739
E1 (5)	T	C	C	T	MF034655
E2 (3)	T	T	T	C	MF034657
E3 (2)	T	T	T	C	MF034658

**Table 5:** Intra-assemblage substitutions in *tpi*, *gdh* and *bg* sequences from assemblage A

Subtype (number)	Nucleotide positions and substitutions					GenBank ID
	58	122	255	269	307	
*bg*						
Ref. sequence	C	C	A	A	C	KT728529
A1 (4)	T	C	A	A	T	KY989576
A2 (3)	C	C	A	A	C	KY989577
A3 (1)	C	T	A	G	C	KY989578
A4 (1)	C	C	G	A	C	KY989579
	8	120	180	240	300	
*tpi*						
Ref. sequence	C	A	G	A	A	KU382249
A (5)	C	A	G	A	A	KY989583
	56	120	180	240	300	
*gdh*						
Ref. sequence	T	T	C	C	G	JF792402
A (1)	T	T	C	C	G	MF034656

**Table 6:** Multilocus characterization of *Giardia* isolates based on the *bg*, *tpi* and *gdh* genes

Isolate	Genotype or subtype			MLG type
	*bg*	*tpi*	*gdh*	
ZZF6, LTB12, LTD2	E	E1	–^a^	–
HZB5, HZB11, HZB19	E	E2	E1	MLGE1
HZB20, HZC9, LTA7	E	E2	–^a^	–
LTA4, LTA18	E	E2	E2	MLGE2
LTB7, LTD10	E	E1	E1	MLGE3
LTB10	E	E3	–^a^	–
LTD6	E	A	–_a_	Mixed
LTD9, LTE14, LTE15	A1	A	–^a^	–
YLA4	A2	–^a^	A	–
YLA21	E	–^a^	E2	–
YLA24	E	–^a^	E3	–
YLA35	A2	A	–^a^	–
YLD13	E	E1	E3	MLGE4

^a^No amplification

## Conclusions

The prevalence and MLGs of *G. duodenalis* in pigs from Shaanxi Province, northwestern China, were investigated in the present study. The total prevalence of *G. duodenalis* infection was 8% and the highest infection rate was observed in sow. Assemblage analysis indicated the presence of the animal-specific assemblage E and the potentially zoonotic assemblage A. Genetic diversity was found within both assemblages, and four assemblage E MLGs were discovered. To the best of our knowledge, this is the first investigation of *G. duodenalis* MLGs in pigs. The findings in our study provided basic data for understanding the molecular epidemiology of *G. duodenalis* in pigs, and highlighted the significance of multilocus genotyping for unraveling the intricate molecular epidemiology of giardiasis in animals and impact on livestock economics and human health. However, there were some limitations to the sampling strategies and study methodologies in our study. For example, no statistical analysis of prevalence in different seasons was conducted in our study. Therefore, additional factors should be included in future studies to accurately determine the infection status of *G. duodenalis* in pigs in Shaanxi Province as well as other geographical locations.

## Abbreviations

*Bg*: β-giardin
*Gdh*: glutamate dehydrogenase
MLGs: multilocus genotyping
SSU rRNA: small subunit ribosomal RNA
*Tpi*: triosephosphate isomerase
